# Secure dynamic multiparty quantum private comparison

**DOI:** 10.1038/s41598-019-53967-9

**Published:** 2019-11-28

**Authors:** Hussein Abulkasim, Ahmed Farouk, Safwat Hamad, Atefeh Mashatan, Shohini Ghose

**Affiliations:** 1 Ted Rogers School of Information Technology Management, Ryerson University, Toronto, Canada; 2 Faculty of Science, The New Valley University, El-kharga, Egypt; 3 Faculty of Science, South Valley University, Qena, 83523 Egypt; 4 Department of Physics and Computer Science, Wilfrid Laurier University, Waterloo, Canada; 5 Perimeter Institute for Theoretical Physics, Waterloo, Canada; 6 Faculty of Computer and Information Sciences, Ain Shams University, Cairo, 11566 Egypt

**Keywords:** Computer science, Information theory and computation

## Abstract

We propose a feasible and efficient dynamic multiparty quantum private comparison protocol that is fully secure against participant attacks. In the proposed scheme, two almost-dishonest third parties generate two random keys and send them to all participants. Every participant independently encrypts their private information with the encryption keys and sends it to the third parties. The third parties can analyze the equality of all or some participants’ secrets without gaining access to the secret information. New participants can dynamically join the protocol without the need for any additional conditions in the protocol. We provide detailed correctness and security analysis of the proposed protocol. Our security analysis of the proposed protocol against both inside and outside attacks proves that attackers cannot extract any secret information.

## Introduction

The pioneering work of Bennett and Brassard^1^ laid the groundwork for the rapidly growing field of quantum cryptography and quantum communication. Subsequently, various quantum protocols have been proposed including Shor’s algorithm for factoring^2–4^, quantum teleportation^5–9^, superdense coding^10–13^, quantum secure direct communication^14–16^, quantum secret sharing^17–21^, quantum dialogue^22,23^ and quantum key agreement^24,25^. In 1982, the millionaires’ problem was proposed as a possible application of secure multiparty computing^26^, the goal is for two parties to compare their wealth and learn who is wealthier without revealing any extra data about the other’s wealth. In 2001, an efficient and fair solution to the socialist millionaires’ problem was proposed^27^. Furthermore, a solution for the socialist millionaires’ problem based on homomorphic encryption in a semi-honest environment was discussed^28^. Lo^29^ proved that the task of secure two-party computation is unachievable even with quantum cryptography^29^. Therefore, a quantum private comparison (QPC) protocol for comparing the equality of information with the help of a third party (TP) was proposed^30^. Furthermore, Hung *et al*.^31^ proposed a secure QPC protocol with two almost-dishonest TPs. In general, there are four common levels of TP’s trustworthiness^32,33^: (1) TP is fully honest. In this circumstance, the participants only send their encrypted secrets to the TP. The TP then compares the private information of the participants and announces the final result. This situation is surely ideal, but finding a fully honest TP in the real world could be challenging. (2) TP is dishonest such that all participants cannot trust the TP. This assumption is equivalent to the standard two-party QPC protocols without a TP, whose insecurity was proved by Lo^29^. (3) TP is semi-honest. Under this circumstance, the participants can partially trust the TP. The TP honestly executes the required processes and may eavesdrop on participants’ private information using passive attacks^31^. (4) TP is almost-dishonest. This situation, which is more reasonable, assumes that the participants can partially trust the TP, and the TP may perform any active attack while executing the protocol, except conspiring with dishonest participants^31^. In general, QPC protocols can be used for novel and existing applications, including quantum voting^34,35^, quantum bidding^36^, and quantum auctions^37–39^.

Chang *et al*. proposed the first multiparty quantum private comparison (MQPC) protocol for comparing the equality of secrets of any two parties among *M* participants^40^. The protocol used GHZ states as a quantum resource. Subsequently, a novel QPC protocol that included the support of a semi-honest TP and used d-dimensional entangled photons was proposed^41^. An MQPC protocol based on entanglement swapping of Bell states was subsequently presented^42^. This scheme used the one-way hash function to address information leakage issue and to encrypt secret information between the communicating parties. A pioneering *M*-participant QPC protocol that addressed the possibility of a dishonest TP collaborating with participants was discussed^43^. Furthermore, a novel MQPC protocol with a semi-honest TP that used entanglement swapping of d-level states and a unitary operation to encrypt the participants’ secrets was proposed^44^. Then, Hung *et al*.^31^ presented a QPC protocol consisting of two third-parties in which one is malicious and the other is almost dishonest was presented. A multi-user QPC protocol that employs both scattered preparation operation and one-way convergent transmission operation of quantum states was also proposed^45^, where two participants can compare their secrets with the support of the remaining participants using the polarization and spatial-mode degrees of freedom of photons to transmit information. Liu *et al*.^46^ proposed a QPC protocol in which any participant can join dynamically to participate in the comparison of *M* participants.

These quantum private comparison protocols still suffer from low efficiency and an inadequate level of security. Therefore, this work proposes a feasible, efficient, and secure dynamic multiparty quantum private comparison protocol (DMQPC) that uses single-photons to encode and send encrypted information. Our proposed scheme has several important features. First, dishonest participants cannot individually or jointly attack the scheme to gain any private or secret information since every participant independently encrypts and transmits secret information to two TPs without the involvement or assistance of other participants. Second, our protocol is dynamic and flexible such that multiple participants can join or leave the protocol and the two TPs can successfully compare the encrypted information of any subset of *M* participants. Third, the participants only generate and transmit single photons, and the two TPs generate single photons and perform single-photon measurements. Hence, the cost of the deployed quantum devices and the employed quantum operations is reduced, and the efficiency of the proposed protocol is increased. Finally, the communication cost is significantly reduced since the proposed protocol can be executed in a variable number of rounds. We describe our scheme and provide proofs and illustrative examples in the following sections. Section 2 introduces the proposed DMQPC protocol. Section 3 verifies the correctness of the proposed scheme. The security analysis is presented in Section 4. Section 5 discussed the efficiency of the scheme and comparisons to some previous protocols. We show that our scheme is more feasible, efficient, secure and flexible compared to other protocols. Section 6 introduces comparisons to some existing QPC protocols. A summary and conclusion is presented in Section 7.

## The Proposed DMQPC Protocol

Here, we will discuss the DMQPC protocol for three different scenarios, namely two-party QPC with two rounds, DMQPC with two rounds and DMQPC with B-block. Before the comparison of data, there are two main processes: (1) validation check process; (2) the initial preparation and encryption process. The two processes are similar in the three scenarios. So, they will be described in detail only for two-party QPC with two rounds.

### Two-party QPC with two rounds

Suppose that Alice and Bob intend to compare the equality of their secrets *X* and *Y*, respectively, with the help of two almost-dishonest *TPs*. The binary representation of *X* in *F*_2*n*_ is (*x*_0_, *x*_1_, ..., *x*_*n*−1_), and the binary representation of *Y* in *F*_2*n*_ is (*y*_0_, *y*_1_, ..., *y*_*n*−1_) where *X*_*i*_, *Y*_*i*_ ∈ {0, 1}^*n*^ and *n* ≥ 2 is the number of secret bits. In general, a protocol with two TPs has many advantages such as: (1) improving load balance performance since we can distribute the workload to two TPs (servers) instead of only one; (2) increasing availability that ensures continuity of communication; (3) ensuring security since one TP can monitor the performance of the other one^31^. The idea of adopting two TPs to execute the comparison task in QPC was first suggested by Hung *et al*.^31^. In our work, the advantage of using two TPs is that one can generate two independent random keys by two different TPs. More specifically, the first third-party (*TP*_1_) computes the comparison result of the first round. The second third-party (*TP*_2_) computes the comparison result of the second-round. Both *TP*_1_ and *TP*_2_ prepare a random secret key and send it to both Alice and Bob.

#### Validation check process

Firstly, *X* and *Y* must have the same length. Secondly, to correctly execute the proposed QPC protocol, secret data must be checked as follows; If the length of *X*(*Y*) is odd, then Alice (Bob) must replace the last bit with two bits;

1 $$\{\begin{array}{c}0\to 00\\ 1\to 10\end{array}$$

#### Initial Preparation and Encryption Process

*TP*_1_ and *TP*_2_ prepare two random secret keys ${{\rm{K}}}_{rand}^{TP1}$ and ${{\rm{K}}}_{rand}^{TP2}$, respectively, and send them through quantum channels to both Alice and Bob^16,47^. Alice and Bob compute ${K}_{rand}={{\rm{K}}}_{rand}^{TP1}\oplus {{\rm{K}}}_{rand}^{TP2}$, where $|{K}_{rand}|=|{{\rm{K}}}_{rand}^{TP1}|=|{{\rm{K}}}_{rand}^{TP2}|=|X|=|Y|$. Then Alice and Bob split *K*_*rand*_ into two equal parts ${K}_{rand}^{1}$ and ${K}_{rand}^{2}$, where *K*_*rand*_ ∈ {0, 1}^*n*^ and ${K}_{rand}^{1},{K}_{rand}^{2}\in {\{0,1\}}^{\frac{n}{2}}$. To reduce the communication cost, Alice also divides *X* into two equal parts *X*_*part*_1_ and *X*_*part*_2_. Alice then computes

2 $${X}_{1}={K}_{rand}^{1}\oplus {X}_{part\_1},$$

3 $${X}_{2}={K}_{rand}^{2}\oplus {X}_{part\_2}.$$

The encrypted parts *X*_1_ and *X*_2_ can be represented as follows.

4 $${X}_{1}=\{{x}_{1,0},{x}_{1,1},\,\mathrm{..}.,\,{x}_{1,(\frac{n}{2}-1)}\},$$

5 $${X}_{2}=\{{x}_{2,\frac{n}{2}},{x}_{2,(\frac{n}{2}+1)},\,\mathrm{..}.,\,{x}_{2,(n-1)}\},$$

where *X*_1_ and *X*_2_ are the first and second parts of *X* encrypted with ${K}_{rand}^{1}$ and ${K}_{rand}^{2}$, respectively. Similarly, Bob computes *Y*_1_ and *Y*_2_ just as Alice does.

6 $${Y}_{1}=\{{y}_{1,0},{y}_{1,1},\,\mathrm{..}.,\,{y}_{1,(\frac{n}{2}-1)}\},$$

7 $${Y}_{2}=\{{y}_{2,\frac{n}{2}},{y}_{2,(\frac{n}{2}+1)},\,\mathrm{..}.,\,{y}_{2,(n-1)}\},$$

where *Y*_1_ and *Y*_2_ are the first and second parts of *Y* encrypted with ${K}_{rand}^{1}$ and ${K}_{rand}^{2}$, respectively. Also, we have *X*_12_ = *X*_1_ ⊕ *X*_2_ and *Y*_12_ = *Y*_1_ ⊕ *Y*_2_. Here, ⊕ is the exclusive-OR operation.

As shown in Table 1, Alice generates new encoded parts ${X}_{1}^{\text{'}}$ and ${X}_{12}^{\text{'}}$ from *X*_1_, *X*_2_, and *X*_12_ according to the following rule: If the bit value of *X*_1_ = *X*_12_ = 0(*X*_1_ = *X*_12_ = 1) then $\,{X}_{1}^{\text{'}}={X}_{12}^{\text{'}}=1\,({X}_{1}^{\text{'}}={X}_{12}^{\text{'}}=0)$. Otherwise, ${X}_{1}^{\text{'}}={X}_{1}$ and ${X}_{12}^{\text{'}}={X}_{12}$, where ${X}_{1}^{\text{'}}$ and ${X}_{12}^{\text{'}}$ are updated parts of *X*_1_ and *X*_12_. The purpose of this process is to relate the secret message parts to each other so that we can reduce the communication cost. That is to say, it is possible to only compare one part of the secret messages in some situations to get the final result.

**Table 1:** Illustration of the proposed technique for preparing ${X}_{1}^{\text{'}}$ and ${X}_{12}^{\text{'}}$.

*X*_1_		*X*_2_		*X*_12_ = *X*_1_ ⊕ *X*_2_		${{\boldsymbol{X}}}_{{\bf{1}}}^{\text{'}}$		${{\boldsymbol{X}}}_{{\bf{12}}}^{\text{'}}$	
*x*_1,0_	0	${x}_{2,\frac{n}{2}}$	0	${x}_{\frac{n}{2}}$	0	${x}_{1,0}^{\text{'}}$	1	${x}_{\frac{n}{2}}^{\text{'}}$	1
*x*_1,1_	0	${x}_{2,(\frac{n}{2}+1)}$	1	${x}_{(\frac{n}{2}+1)}$	1	${x}_{1,1}^{\text{'}}$	0	${x}_{(\frac{n}{2}+1)}^{\text{'}}$	1
*x*_1,2_	1	${x}_{2,(\frac{n}{2}+2)}$	0	${x}_{(\frac{n}{2}+2)}$	1	${x}_{1,2}^{\text{'}}$	0	${x}_{(\frac{n}{2}+2)}^{\text{'}}$	0
…	…	…	…	…	…	…	…	…	…
${x}_{1,(\frac{n}{2}-1)}$	1	*x*_2,(*n*−1)_	1	*x*_(*n*−1)_	0	${x}_{1,(\frac{n}{2}-1)}^{\text{'}}$	1	${x}_{(n-1)}^{\text{'}}$	0

From Table 1, we can get the sequences ${X}_{1}^{\text{'}}$, *X*_12_, and ${X}_{12}^{\text{'}}$, with length $\frac{n}{2}$:

8 $${X}_{1}^{\text{'}}=\{{x}_{1,0}^{\text{'}},{x}_{1,1}^{\text{'}},\,\ldots ,\,{x}_{1,(\frac{n}{2}-1)}^{\text{'}}\}$$

9 $${X}_{12}=\{{x}_{\frac{n}{2}},\,{x}_{(\frac{n}{2}+1)},\,\,\ldots ,\,\,{x}_{(n-1)}\},$$

10 $${X}_{12}^{\text{'}}=\{{x}_{\frac{n}{2}}^{\text{'}},{x}_{(\frac{n}{2}+1)}^{\text{'}},\,\ldots ,\,{x}_{(n-1)}^{\text{'}}\}.$$

Alice uses the XOR function to encrypt *X*_1_ with ${X}_{1}^{\text{'}}$ getting *C*_*a*1_,

11 $${C}_{a1}={X}_{1}\oplus {X}_{1}^{\text{'}}=\{({x}_{1,0}\oplus {x}_{1,0}^{\text{'}}),({x}_{1,1}\oplus {x}_{1,1}^{\text{'}}),\,\mathrm{..}.,\,\{{x}_{1,(\frac{n}{2}-1)}\oplus {x}_{1,\,(\frac{n}{2}-1)}^{\text{'}}\},$$

Similarly, Bob performs the same processes as Alice does,

12 $${C}_{b1}={Y}_{1}\oplus {Y}_{1}^{\text{'}},$$

Alice computes *X*_12_ = *X*_1_ ⊕ *X*_2_:

13 $${X}_{12}=\{({x}_{1,0}\oplus {x}_{2,\frac{n}{2}}),({x}_{1,1}\oplus {x}_{2,(\frac{n}{2}+1)}),\,\mathrm{..}.,\,({x}_{1,(\frac{n}{2}-1)}\oplus {x}_{2,(n-1)})\}.$$

Bob also computes *Y*_12_ = *Y*_1_ ⊕ *Y*_2_:

14 $${Y}_{12}=\{({y}_{1,0}\oplus {y}_{2,\frac{n}{2}}),({y}_{1,1}\oplus {y}_{2,(\frac{n}{2}+1)}),\,\mathrm{..}.,\,({y}_{1,(\frac{n}{2}-1)}\oplus {y}_{2,(n-1)})\}.$$

In our protocol, we have three options to compute and announce the comparison result. The first option would be for *TP*_1_ to compute and announce (in the first and second rounds) the comparison result. The second option would be for *TP*_2_ to compute and announce the comparison result. These two options can be used when availability of at least one *TP* is the most important requirement. The third option would be for the two *TPs* to collaborate to compute and announce the final result. The steps for executing the two rounds to compare the equality of parties’ secrets are similar in the three options. The choice of which of the three options to use depends on whether the priority is availability, workload or security. The two rounds are described as follows.

#### The first-round

***Step 1***. *TP*_1_ asks Alice and Bob to prepare C_a1_ = X_1_ ⊕ ${{\rm{X}}}_{1}^{\text{'}}$ and C_b1_ = Y_1_ ⊕ ${{\rm{Y}}}_{1}^{\text{'}}$, respectively.

***Step 2***. Alice prepares a sequence of $\frac{n}{2}$ single photons, called *S*_*a*1_, corresponding to *C*_*a*1_ in the Z-basis {|0〉, |1〉} or the X-basis $\{|+\rangle =\frac{1}{\sqrt{2}}(|0\rangle +|1\rangle ),|-\rangle =\frac{1}{\sqrt{2}}(|0\rangle -|1\rangle )\}$.

***Step 3***. For the eavesdropping check, Alice randomly prepares a sequence of decoy photons *l*_*a*1_ in one of the states {|0〉, |1〉, |+〉, |−〉}. At random positions, she inserts *l*_*a*1_ into *S*_*a*1_ producing a new sequence ${S}_{a1}^{\text{'}}$. Then, Alice transmits ${S}_{a1}^{\text{'}}$ to the *TP*_1_.

***Step*** **4**. Alice announces the random positions and the measurement bases of *l*_*a*1_ to *TP*_1_ for performing single photon measurements. *TP*_1_ then reveals the measurement outcomes. Hence, *TP*_1_ and Alice analyze the error rate. If the rate is higher than a predetermined threshold, then they terminate the protocol and restart the process again. Otherwise, *TP*_1_ discards *l*_*a*1_ from ${S}_{a1}^{\text{'}}$ and extracts *S*_*a*1_. Then *TP*_1_ can restore *C*_*a*1_, where *S*_*a*1_ represents *C*_*a*1_.

***Step 5***. Bob and *TP*_1_ perform the same *Steps 2–4* as Alice and *TP*_1_ to send *C*_*b*1_ to *TP*_1_.

***Step 6***. *TP*_1_ performs a comparison between the first part of Alice’s and Bob’s secrets by computing *R*_1_ = *C*_*a*1_ ⊕ *C*_*b*1_. If *R*_1_ = 0, this indicates that *X* and *Y* may be equal. In this case, they move to the next round to check whether Alice’s and Bob’s secrets are equal or not. Otherwise, *X* and *Y* are not equal, so there is no need to continue to the second-round to check the equality of the second parts.

#### The second-round

***Step 7***. *TP*_1_ informs *TP*_2_ that the first-round comparison result may be equal. Then *TP*_2_ asks Alice and Bob to prepare *X*_12_ and *Y*_12_, respectively.

***Step 8***. Alice and Bob perform the same processes described in *Steps 2–4* to send *X*_12_ and *Y*_12_ to *TP*_2_.

***Step 9***. *TP*_2_ computes *R*_2_ = *X*_12_ ⊕ *Y*_12_. If *R* = *R*_1_ + *R*_2_ = 0 then *X* and *Y* are equal. Otherwise, *X* and *Y* are not equal. A detailed example to check the equality of *X* = {001100110010} and *Y* = {011100110010} is shown in Tables 2 and 3.

**Table 2:** Illustration of preparation of encrypted secrets for two participants.

The private information		*X* = {001100110010}	*Y* = {011100110010}
Random keys		${{\rm{K}}}_{rand}^{TP1}=\{010110010110\}$, ${{\rm{K}}}_{rand}^{TP2}=\{111111010010\}$	
		${K}_{rand}={{\rm{K}}}_{rand}^{TP1}\oplus {{\rm{K}}}_{rand}^{TP2}=\{101001000100\}$, ${K}_{rand}^{1}=\{101001\}$, ${K}_{rand}^{2}=\{000100\}$	
Validity check	Length check for equality	*X*_*length* = *Y*_*length* = 12	
	Length check for 2 blocks	$\frac{12}{2}=6$	
Initial preparation		*X*_*part*_1_ = {001100}, *X*_*part*_2_ = {110010}, ${K}_{rand}^{1}=\{101001\}$, ${K}_{rand}^{2}=\{000100\}$.	*Y*_*part*_1_ = {011100}, *Y*_*part*_2_ = {110010}, ${K}_{rand}^{1}=\{101001\}$, ${K}_{rand}^{2}=\{000100\}$.
Encryption		${X}_{1}={K}_{rand}^{1}\oplus {X}_{part\_1}$, ${X}_{2}={K}_{rand}^{2}\oplus {X}_{part\_2}$, *X*_1_ = {100101}, *X*_2_ = {110110}	${Y}_{1}={K}_{rand}^{1}\oplus {Y}_{part\_1}$, ${Y}_{2}={K}_{rand}^{2}\oplus {Y}_{part\_2}$, *Y*_1_ = {110101}, *Y*_2_ = {110110}.
Encoding If ${X}_{1}={X}_{12}=0\,({X}_{1}={X}_{12}=1)$ then $\,{X}_{1}^{\text{'}}={X}_{12}^{\text{'}}=1\,({X}_{1}^{\text{'}}={X}_{12}^{\text{'}}=0)$. Else, ${X}_{1}^{\text{'}}={X}_{1}$ & ${X}_{12}^{\text{'}}={X}_{12}$ The same process for *Y*			
		${X}_{1}=100101$, ${X}_{1}^{\text{'}}=101100$, ${X}_{12}=010011$.	${Y}_{1}=110101$, ${Y}_{1}^{\text{'}}=111100$, ${Y}_{12}=000011$.
Compute ${C}_{a1}={X}_{1}\oplus {X}_{1}^{\text{'}}$, ${X}_{12}={X}_{1}\oplus {X}_{2}$, & ${C}_{b1}={Y}_{1}\oplus {Y}_{1}^{\text{'}}$, ${Y}_{12}={Y}_{1}\oplus {Y}_{2}$.		${C}_{a1}=\{001001\}$, ${X}_{12}=\{010011\}$.	${C}_{b1}=\{001001\}$, ${Y}_{12}=\{000011\}$.

**Table 3:** Illustration of the equality check of *X* and *Y*.

Round 1	*Alice*	*TP*_1_	*Bob*
Step 1: Preparation 〈*Alice*〉	Prepares ${C}_{a1}=\{001001\}$ in *Z*-basis or *X*-basis		
Steps 2&4: Eavesdropping check 〈*Alice*, *TP*_1_〉	$error\,rate\, < specified\,Threshold$, *TP*_1_ obtains ${C}_{a1}$. Else, the communication process is terminated.		
Step 5: Preparation 〈*Bob*〉			Prepares ${C}_{b1}=\{001001\}$, in *Z*-basis or *X*-basis
Step 5: Eavesdropping check 〈*Bob*, *TP*_1_〉		$error\,rate\, < specified\,Threshold$, *TP*_1_ obtains ${C}_{b1}$. Else, the communication process is terminated.	
Step 6: Check the equality		If ${R}_{1}={C}_{a1}\oplus {C}_{b1}\ne 0$; ${X}_{1}\ne {Y}_{1}$, $X\ne Y$. The protocol will terminate and no need for a second-round. Otherwise, they continue to *Round 2*.	
**Round 2**	***Alice***	*TP*_**2**_	***Bob***
Step 7: Preparation 〈*Alice*〉	Prepares ${X}_{12}=\{010011\}$ in *Z*-basis or *X*-basis		
Step 8: Eavesdropping check 〈*Alice*, *TP*_2_〉	*error rate* < *specified Threshold*, *TP*_2_ obtains *X*_12_. Otherwise, the communication process is terminated.		
Step 7: Preparation 〈*Bob*〉			Prepares ${Y}_{12}=\{000011\}$ in *Z*-basis or *X*-basis
Step 8: Eavesdropping check 〈*Bob*, *TP*_2_〉		*error rate* < *specified Threshold*, *TP*_2_ obtains *Y*_12_. Otherwise, the communication process is terminated.	
Step 9: Check the equality		If ${R}_{2}={X}_{12}\oplus {Y}_{12}=0$; *X* = *Y*. Otherwise, *X* ≠ *Y*.	

### Adding new participants

One of the main features of this protocol is the ease of joining of one or more participants. Without loss of generality, suppose a new participant called Charlie want to joint the old participants (Alice and Bob). The steps for adding a new participant are described as follows.

#### The first-round

***Step 1***. Charlie asks *TP*_1_ and *TP*_2_ to join the protocol.

***Step 2***. *TP*_1_ asks Charlie to prepare ${{\rm{C}}}_{c1}={{\rm{Z}}}_{1}\oplus {{\rm{Z}}}_{1}^{\text{'}}$ using the same protocol as Alice and Bob to prepare C_*a*1_ and C_*b*1_, respectively.

***Step 3***. Charlie prepares a sequence of $\frac{n}{2}$ single photons, called *S*_*c*1_, corresponding to *C*_*c*1_ in the Z-basis {|0〉, |1〉} or the X-basis $\{|\,+\,\rangle =\frac{1}{\sqrt{2}}(|0\rangle +|1\rangle ),|\,-\,\rangle =\frac{1}{\sqrt{2}}(|0\rangle -|1\rangle )\}$.

***Step 4***. For eavesdropping check, Charlie randomly prepares a sequence of decoy photons *l*_*c*1_ in one of the states {|0〉, |1〉, |+〉, |−〉}. At random positions, he inserts *l*_*c*1_ into *S*_*c*1_ producing a new sequence ${S}_{c1}^{\text{'}}$. Then, Charlie transmits ${S}_{c1}^{\text{'}}$ to the *TP*_1_.

***Step 5***. Upon receiving ${S}_{c1}^{\text{'}}$, Charlie announces the random positions and the measurement bases of *l*_*c*1_ to *TP*_1_ for performing single photon measurements. *TP*_1_ then reveals the measurement outcomes. Hence, *TP*_1_ and Charlie analyze the error rate. If the rate is higher than a predetermined threshold, then they terminate the protocol and restart the process again. Otherwise, *TP*_1_ discards *l*_*c*1_ from ${S}_{c1}^{\text{'}}$ and extracts *S*_*c*1_. Then *TP*_1_ can restore *C*_*c*1_.

***Step 6***. *TP*_1_ performs a comparison between the first part of Alice’s, Bob’s, and Charlie’s secrets by computing *R*_1_ = (*C*_*a*1_ ⊕ *C*_*b*1_) + (*C*_*b*1_ ⊕ *C*_*c*1_). If *R*_1_ = 0, this indicates that *X*, *Y*, and *Z* may be equal. In this case, they move to the next round to check whether Alice’s, Bob’s, and Charlie’s secrets are equal or not. Otherwise, *X*, *Y*, and *Z* are not equal, so there is no need to continue to the second-round to check the equality of the second parts.

#### The second-round

***Step 7***. *TP*_1_ informs *TP*_2_ that the first-round comparison result may be equal. Then *TP*_2_ asks Charlie to prepare *Z*_12_ using the same protocol as Alice and Bob to prepare X_12_ and Y_12_, respectively.

***Step 8***. Charlie performs the same processes described in *Steps 3–4* to send *Z*_12_ to *TP*_2_.

***Step 9***. *TP*_2_ computes *R*_2_ = (*X*_12_ ⊕ *Y*_12_) + (*Y*_12_ ⊕ *Z*_12_). If *R* = *R*_1_ + *R*_2_ = 0, *TP*_2_ announces to Alice, Bob, and Charlie that *X*, *Y*, and *Z* are equal. Otherwise, *X*, *Y*, and *Z* are not equal.

### Deleting old participants

Without loss of generality, suppose we have three participants Alice, Bob, and Charlie. *TP*_1_ and *TP*_2_ are allowed to delete one or more participants (e.g., Charlie) for several reasons. For example, they may want to compare just Bob’s and Alice’s private information. The detailed steps for deleting Charlie are as follows.

#### The first-round

***Step 1***. *TP*_1_ and *TP*_2_ agree to delete Charlie. *TP*_1_ then discards *C*_*c*1_.

***Step 2***. *TP*_1_ updates the comparison process, to be only between Alice and Bob, *TP*_1_ then recomputes *R*_1_. In that case, *TP*_1_ computes and considers the result of *R*_1_ = *C*_*a*1_ ⊕ *C*_*b*1_ instead of *R*_1_ = (*C*_*a*1_ ⊕ *C*_*b*1_) + (*C*_*b*1_ ⊕ *C*_*c*1_). If the result of *R*_1_ = 0, this indicates that *X* and *Y* may be equal. In this case, they move to the next round to check whether Alice’s and Bob’s secrets are equal or not. Otherwise, *X* and *Y* are not equal and the final result is announced.

#### The second-round

***Step 3***. *TP*_1_ informs *TP*_2_ that the first-round comparison result of Alice’s and Bob’s secrets may be equal.***Step 4***. *TP*_2_ discards the encrypted information of Charlie (*Z*_12_) and only considers the private information of Alice and Bob, that is, *X*_12_ and *Y*_12_, respectively.

***Step 5***. *TP*_2_ computes and considers *R*_2_ = *X*_12_ ⊕ *Y*_12_ instead of *R*_2_ = (*X*_12_ ⊕ *Y*_12_) + (*Y*_12_ ⊕ *Z*_12_). If *R* = *R*_1_ + *R*_2_ = 0 then *X* and *Y* are equal. Otherwise, *X* and *Y* are not equal.

### Multi-party QPC with two rounds

**Figure 1:**
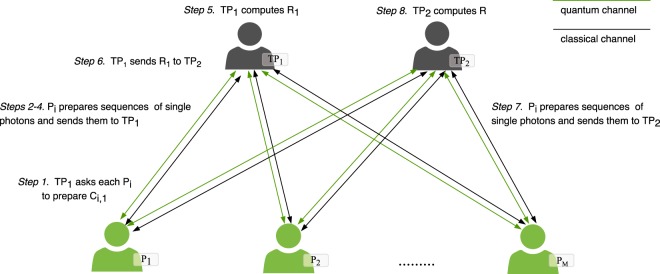
The proposed DMQPC protocol for *M* participants.

The proposed two-party QPC protocol is easy to extend to *M* participants (see Fig. 1). In this scenario, there are *M* participants *P*_*i*_ (*i* = 1, 2, ..., *M*), and each of them has secret information ${X}_{i}^{\ast }$ with length *n*. Firstly, participants check the validity of their secrets according to the validation check process. After they make sure that their secrets are valid for applying the proposed protocol, *TP*_1_ and *TP*_2_ send two random secret keys (${{\rm{K}}}_{rand}^{TP1}$ and ${{\rm{K}}}_{rand}^{TP2}$) with length *n* to all participants. *P*_*i*_ then perform the initial preparation and encryption process as shown in Eqs. (2–5) for producing ${X}_{i,1}^{\ast }$ and ${X}_{i,2}^{\ast }$. From Table 1, each participant gets the sequences ${X}_{i,1}^{\ast }$ and ${X}_{i,2}^{\ast }$, with length $\frac{n}{2}$ for each sequence. Also, each participant computes *C*_*i*,1_ = ${X}_{i,1}^{\ast }$ ⊕ ${X}_{i,1}^{^{\prime} }$. Now each participant has completed preparing encrypted secrets, and they are ready for checking the equality of their secrets using the QPC protocol.

#### The first-round

***Step 1***. *TP*_1_ asks each participant to prepare *C*_*i*,1_.

***Step 2***. *P*_*i*_ prepares a quantum sequence containing $\frac{n}{2}$ single photons corresponding to *C*_*i*,1_ (i.e. *S*_*i*,1_) in the Z-basis {|0〉, |1〉} or X-basis $\{|+=\frac{1}{\sqrt{2}}(|0\rangle +|1\rangle ,|\,-\,\rangle =\frac{1}{\sqrt{2}}(|0\rangle -|1\rangle )\}$.

***Step 3***. For the eavesdropping check, *P*_*i*_ randomly prepares a sequence of decoy photons *l*_*i*,1_ in one of the states {|0〉, |1〉, |+〉, |−〉}. At random positions, *P*_*i*_ inserts *l*_*i*,1_ into *S*_*i*,1_ producing a new sequence ${S}_{i,1}^{\text{'}}$. Then, *P*_*i*_ sends ${S}_{i,1}^{\text{'}}$ to the *TP*_1_.

***Step 4***. Upon receiving ${S}_{i,1}^{\text{'}}$, *P*_*i*_ announces the random positions and the measurement bases of *l*_*i*,1_ to *TP*_1_ for performing single photon measurements. *TP*_1_ then announces the measurement outcomes. *TP*_1_ and *P*_*i*_ analyze the error rate. If the rate is higher than a predetermined threshold, they terminate the communication and restart the process again. Otherwise, *TP*_1_ discards *l*_*i*,1_ from ${S}_{i,1}^{\text{'}}$ and extracts *S*_*i*,1_. Then the *TP*_1_ can restore *C*_*i*,1_, where *S*_*i*,1_ represents *C*_*i*,1_.

***Step 5***. *TP*_1_ performs a comparison of the first part of *P*_*i*_’s secret, where for *M* = 3

15 $${R}_{1}=({C}_{1,1}\oplus {C}_{2,1})+({C}_{2,1}\oplus {C}_{3,1}),$$

For *M* > 3

16 $${R}_{1}=({C}_{1,1}\oplus {C}_{2,1})+({C}_{2,1}\oplus {C}_{3,1})+\cdots +({C}_{M-1,1}\oplus {C}_{M,1}).$$

If *R*_1_ = 0, ${X}_{1}^{\ast },\,{X}_{2}^{\ast },\,\ldots ,\,{X}_{M}^{\ast }$ may be equal. Hence, they move to the next round to compute the comparison check of *X*_*i*,12_. Otherwise, ${X}_{1}^{\ast },{X}_{2}^{\ast },\ldots ,\,{X}_{M}^{\ast }$ are not equal. Then it is not necessary to execute the second-round to check the equality of *X*_*i*,12_.

#### The second-round

***Step 6***. *TP*_1_ informs *TP*_2_ that the first-round comparison result may be equal. Then *TP*_2_ asks *P*_*i*_ to prepare *X*_*i*,12_.

***Step 7****. P*_*i*_ performs the same processes as in Steps 2–4 to send *X*_*i*,12_ to *TP*_2_.

***Step 8****. TP*_2_ computes the comparison check of *X*_*i*,12_,

where for *M* = 3

17 $${R}_{2}=({X}_{1,12}\oplus {X}_{2,12})+({X}_{2,12}\oplus {X}_{3,12}),$$

for *M* > 3

18 $${R}_{2}=({X}_{1,12}\oplus {X}_{2,12})+({X}_{2,12}\oplus {X}_{3,12})+\,\cdots \,+({X}_{M-1,12}\oplus {X}_{M,12}),$$

Now, *TP*_2_ can compute *R* = *R*_1_ + *R*_2_ to determine whether ${X}_{1}^{\ast },{X}_{2}^{\ast },\,\ldots ,\,{X}_{M}^{\ast }$ are equal or not. If ${X}_{1}^{\ast },{X}_{2}^{\ast },\,\ldots ,\,{X}_{M}^{\ast }$ are equal. Otherwise, ${X}_{1}^{\ast },{X}_{2}^{\ast },\,\ldots ,\,{X}_{M}^{\ast }$ are not equal. Obviously, it is easy to add or remove any subset of participants to the protocol, where participants independently perform the required processes to prepare their secret for the final step of the protocol. Moreover, *TP*_1_ and *TP*_2_ can easily compare the equality of the secrets of any subset of *M* participants without any additional conditions.

### Multi-Party QPC with B blocks

The secret data can be divided into several blocks (*B*), which could be useful in comparing the equality of big data. Each block contains $\frac{n}{B}$ bits and is executed in two rounds, where $\frac{n}{B}$ is an even number such that,

19 $$2\le \frac{n}{B}\le n\{\begin{array}{c}B\,is\,even\\ \,B\,is\,odd,\,and\,\frac{n}{B}\,is\,even\end{array}.$$

**Figure 2:**
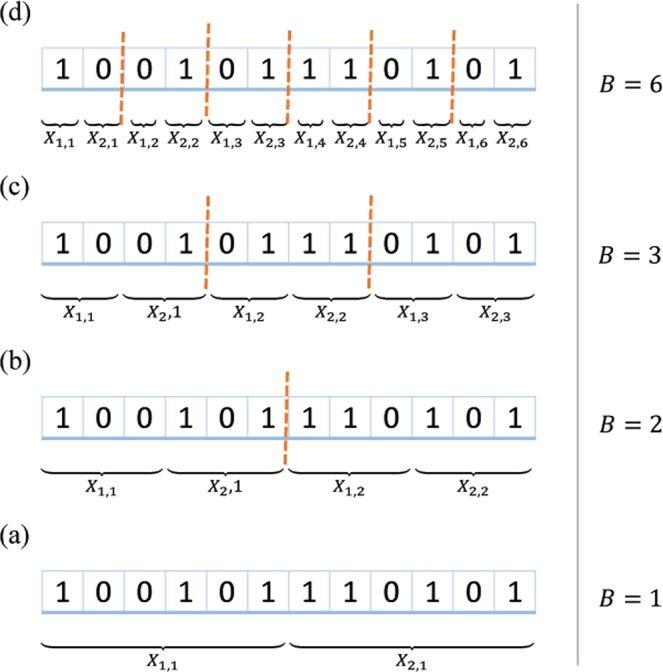
A secret of length 12 can be divided into: (**a**) 1 block divided into two parts and executed in two rounds; (**b**) 2 blocks; (**c**) 3 blocks; (**d**) 6 blocks with two rounds for each block.

Suppose there are *M* participants *P*_*i*_ (*i* = 1, 2, ..., *M*). Each of them has secret information *X*_*i*_ with a length of *n*, and they would like to check the equality of their secrets. Firstly, all participants check the validity of their secrets according to the previously described validation check. After they make sure that their secrets are valid for applying the proposed protocol, *TP*_1_ and *TP*_2_ send two random secret keys (${{\rm{K}}}_{rand}^{TP1}$ and ${{\rm{K}}}_{rand}^{TP2}$) with length *n* to all participants. Based on the length of the secret data (*n*), *TP*_1_ and *TP*_2_ agree with participants on the value of *B* (see Fig. 2). *P*_*i*_ computes ${K}_{rand}={{\rm{K}}}_{rand}^{TP1}\oplus {{\rm{K}}}_{rand}^{TP2}$ and divides *K*_*rand*_ into *B* blocks. Each block contains two sub-keys ${{\rm{K}}}_{rand}^{1,\,j}$ and ${{\rm{K}}}_{rand}^{2,\,j}$, where *j* = 1,2, …, *B*.

Subsequently, *P*_*i*_ performs the initial preparations as previously indicated in Eq. (2) and Eq. (3) for generating ${X}_{i,j}^{1}$ and ${X}_{i,j}^{2}$, where *i* = 1, 2, …, *M*. At this point, using Table 1, participants can easily prepare their encrypted secret information producing *C*_*i*,*j*_ and ${X}_{12}^{i,j}$, and are ready to check the equality of their secrets using the following steps.

#### The first-round

***Step 1****. TP*_1_ asks each participant to prepare *C*_*i*,j_.

***Step 2***. *P*_*i*_ prepares a sequence of $\frac{n}{2B}$ single photons for each block, called *S*_*i*,*j*_, corresponding to *C*_*i*,*j*_, in the Z-basis {|0,〉 |1〉} or X-basis $\{|\,+\,\rangle =\frac{1}{\sqrt{2}}(|0\rangle +|1\rangle ),|\,-\,\rangle =\frac{1}{\sqrt{2}}(|0\rangle -|1\rangle )\}$.

***Step 3***. To prevent eavesdropping, *P*_*i*_ randomly prepares a sequence of decoy photons *l*_*i*,*j*_ in one of the states {|0〉, |1〉, |+〉, |−〉}. At random positions, *P*_*i*_ inserts *l*_*i*,*j*_ into *S*_*i*,*j*_ producing a new sequence ${S}_{i,1}^{\text{'}}$. *P*_*i*_ then sends ${S}_{i,1}^{\text{'}}({S}_{i,2}^{\text{'}},\,\ldots ,\,{S}_{i,B}^{\text{'}})$ to *TP*_1_.

***Step 4****.* Upon receiving ${S}_{i,j}^{\text{'}}$, *P*_*i*_ announces the random positions and the measurement bases of *l*_*i*,*j*_ to *TP*_1_ for performing single photon measurements. *TP*_1_ then announces the measurement outcomes. *TP*_1_ and *P*_*i*_ analyze the error rate. For any error rate above a predetermined threshold, they cancel the communication and restart all over again. Otherwise, *TP*_1_ discards *l*_*i*,*j*_ from ${S}_{i,j}^{\text{'}}$ and extracts *S*_*i*,*j*_. *TP*_1_ then can construct *C*_*i*,*j*_, where *S*_*i*,*j*_ represents *C*_*i*,*j*_.

***Step 5***. *TP*_1_ computes the comparison check of *C*_*i*,*j*_, where for *M* = 3

20 $$\begin{array}{rcl}{R}_{1}^{1} & = & ({C}_{1,1}\oplus {C}_{2,1})+({C}_{2,1}\oplus {C}_{3,1})({R}_{1}^{2}=({C}_{1,2}\oplus {C}_{2,2})+({C}_{2,2}\oplus {C}_{3,2}),\,\ldots ,\,{R}_{1}^{B}\\  & = & ({C}_{1,B}\oplus {C}_{2,B})+({C}_{2,B}\oplus {C}_{3,B})).\end{array}$$

For *M* > 3

21 $$\begin{array}{rcl}{R}_{1}^{1} & = & ({C}_{1,1}\oplus {C}_{2,1})+({C}_{2,1}\oplus {C}_{3,1})+\ldots +({C}_{M-1,1}\oplus {C}_{M,1})({R}_{1}^{2}=({C}_{1,2}\oplus {C}_{2,2})\\  &  & +\,({C}_{2,2}\oplus {C}_{3,2})+\ldots +({C}_{M-1,2}\oplus {C}_{M,2}),\,\ldots ,\,{R}_{1}^{B}=({C}_{1,B}\oplus {C}_{2,B})\\  &  & +\,({C}_{2,B}\oplus {C}_{3,B})+\ldots +({C}_{M-1,B}\oplus {C}_{M,B})).\end{array}$$

If ${R}_{1}^{1}=0\,({R}_{1}^{2}=0,\ldots ,{R}_{1}^{B}=0)$, *X*_1_, *X*_2_, …, *X*_*M*_ may be equal, where ${R}_{1}^{B}$is the comparison result of the first round of block number *B* for all participants. Hence, they move to the next round to compute the comparison check of ${X}_{12}^{i,1}$ ${X}_{12}^{i,2},\,\ldots ,\,{X}_{12}^{i,B}$. Otherwise, their secrets are not equal.

#### The second-round

***Step 6***. *TP*_1_ informs *TP*_2_ that the first-round comparison result of the 1*st*−*block* (2 *nd*−*block*, …, *Bth*−*block*) may be equal. Then *TP*_2_ asks *P*_*i*_ to prepare ${X}_{12}^{i,1}$ $({X}_{12}^{i,2},\,\ldots ,\,{X}_{12}^{i,B})$.

***Step 7****. P*_*i*_ performs the same processes as in *Steps 2*–*4* to send ${X}_{12}^{i,1}({X}_{12}^{i,2},\,\ldots ,\,{X}_{12}^{i,B})$ to *TP*_2_.

***Step 8***. *TP*_2_ computes the comparison check of ${X}_{12}^{i,1}({X}_{12}^{i,2},\,\ldots ,\,{X}_{12}^{i,B})$, where for *M* = 3

22 $$\begin{array}{rcl}{R}_{2}^{1} & = & ({X}_{12}^{1,1}\oplus {X}_{12}^{2,1})+({X}_{12}^{2,1}\oplus \,{X}_{12}^{3,1})({R}_{2}^{2}=({X}_{12}^{1,2}\oplus {X}_{12}^{2,2})\\  &  & +\,({X}_{12}^{2,2}\oplus {X}_{12}^{3,2}),\,\ldots ,\,{R}_{2}^{B}=({X}_{12}^{1,B}\oplus {X}_{12}^{2,B})+({X}_{12}^{2,B}\oplus {X}_{12}^{3,B})),\end{array}$$

for *M* > 3

23 $$\begin{array}{rcl}{R}_{2}^{1} & = & ({X}_{12}^{1,1}\oplus {X}_{12}^{2,1})+({X}_{12}^{2,1}\oplus {X}_{12}^{3,1})+\ldots +({X}_{12}^{M-1,1}\oplus {X}_{12}^{M,1})({R}_{2}^{2}=({X}_{12}^{1,2}\oplus {X}_{12}^{2,2})\\  &  & +\,({X}_{12}^{2,2}\oplus {X}_{12}^{3,2})+\ldots +({X}_{12}^{M-1,2}\oplus {X}_{12}^{M,2}),\,\ldots ,\,\,{R}_{2}^{B}=({X}_{12}^{1,B}\oplus {X}_{12}^{2,B})\\  &  & +\,({X}_{12}^{2,B}\oplus {X}_{12}^{3,B})+\ldots +({X}_{12}^{M-1,B}\oplus {X}_{12}^{M,B})).\end{array}$$

If $R={R}_{1}^{1}+{R}_{2}^{1}={R}_{1}^{2}+{R}_{2}^{2}=\ldots ={R}_{1}^{B}+{R}_{2}^{B}=0$, this means that *X*_1_, *X*_2_, …, *X*_*M*_ are equal. Otherwise, *X*_1_, *X*_2_, …, *X*_*M*_ are not equal. Note, participants check the result of the first block (${R}_{2}^{1}$) and if ${R}_{2}^{1}$ = 0 they continue to check the next block and so on until they reach the last block; otherwise, *TP*_2_ announces that the secrets are not equal.

## Correctness

From Table 4, according to our initial preparation and encryption method, for every two bits we get two different encrypted bits, that is to say, we get *C*_*a*1_ = 1 and *X*_12_ = 0 only when *X*_1_ = 0 and *X*_2_ = 0. So, the bit values of *C*_*a*1_ and *X*_12_ together are decisive in determining the bit values of *X*_1_ and *X*_2_. Assume we have two participants Alice and Bob, and each participant has two bits *X* = 00 and *Y* = 10, respectively, and *K*_*rand*_ = 00. Alice computes ${C}_{a1}={X}_{1}\oplus {X}_{1}^{\text{'}}={K}_{rand}^{1}\oplus {X}_{par{t}_{1}}\oplus {X}_{1}^{\text{'}}$ getting 1, and sends it to *TP*_1_. Bob also computes ${C}_{b1}={Y}_{1}\oplus {Y}_{1}^{\text{'}}={K}_{rand}^{1}\,\oplus {Y}_{part\_1}\oplus {Y}_{1}^{\text{'}}$ getting 1, and sends it to *TP*_1_. When *TP*_1_ computes *R*_1_ = *C*_*a*1_ ⊕ *C*_*b*1_ he gets *R*_1_ = 0, which means that the secrets of Alice and Bob may be equal or unequal (note if *R*_1_ = 1, *TP*_1_ announces that the secrets of Alice and Bob are not equal). So, they should move to the second-round to compare *X*_12_ and *Y*_12_.

**Table 4:** All possible encrypted data from two bits according to the initial preparation and encryption method, where ${X}_{1}={K}_{rand}^{1}\oplus {X}_{part\_1}$, ${X}_{2}={K}_{rand}^{2}\oplus {X}_{part\_2}$.

*X*_1_	*X*_2_	*X*_12_	${X}_{1}^{\text{'}}$	${X}_{12}^{\text{'}}$	To be sent to *TP*_1_	To be sent to *TP*_2_
					*C*_*a*1_ = *X*_1_ ⊕ ${X}_{1}^{\text{'}}$	*X*_12_
0	0	0	1	1	1	0
0	1	1	0	1	0	1
1	0	1	0	0	1	1
1	1	0	1	0	0	0

In the second-round, Alice and Bob send ${X}_{12}={X}_{1}\oplus {X}_{2}={K}_{rand}^{1}\,\oplus {X}_{part\_1}\,\oplus {K}_{rand}^{2}\,\oplus {X}_{part\_2}$ and ${Y}_{12}={Y}_{1}\oplus {Y}_{2}={K}_{rand}^{1}\,\oplus {Y}_{part\_1}\,\oplus {K}_{rand}^{2}\,\oplus {Y}_{part\_2}$ to *TP*_2_, respectively. *TP*_2_ computes *R*_2_ = *X*_12_ ⊕ *Y*_12_ = 0 ⊕ 1 getting *R*_2_ = 1. *TP*_1_ then computes *R* = *R*_1_ + *R*_2_ getting *R* = 1, which means that *X* and *Y* are not equal. Thus, *X* and *Y* are equal if and only if *R* = *R*_1_ = *R*_2_ = 0. For example, suppose we have *X* = 0000 and *K*_*rand*_ = 0000. Then *X*_1_ = 00 and *X*_2_ = 00. As shown in Table 5, we must get *C*_*a*1_ = *X*_1_ ⊕ ${X}_{1}^{\text{'}}$ = 11 and *X*_12_ = 00 only when *X*_1_ = 00 and *X*_2_ = 00. Also, if we have *Y* = 0000 and *K*_*rand*_ = 0000, then *Y*_1_ = 00 and *Y*_2_ = 00. Hence, we get *C*_*b*1_ = *Y*_1_ ⊕ ${Y}_{1}^{\text{'}}$ = 11 and *Y*_12_ = 00. Now the two TPs can announce that the two inputs are equal by computing *R* = (*C*_*a*1_ ⊕ *C*_*b*1_) + (*X*_12_ ⊕ *Y*_12_) = 0, which proves the correctness of this protocol. Note that if we proposed that *C*_*a*1_ = *X*_2_ ⊕ ${X}_{2}^{\text{'}}$ and *C*_*b*1_ = *Y*_2_ ⊕ ${Y}_{2}^{\text{'}}$ instead of *C*_*a*1_ = *X*_1_ ⊕ ${X}_{1}^{\text{'}}$ and *C*_*b*1_ = *Y*_1_ ⊕ ${Y}_{1}^{\text{'}}$ respectively, we also get the same correct comparison result.

**Table 5:** All possible encrypted data when *X* contains four bits, and both *X*_1_ and *X*_2_ include two bits, where ${X}_{1}={K}_{rand}^{1}\oplus {X}_{part\_1}$, ${X}_{2}={K}_{rand}^{2}\,\oplus {X}_{part\_2}$.

*X*_1_	*X*_2_	*X*_12_	${X}_{1}^{\text{'}}$	${X}_{12}^{\text{'}}$	To be sent to *TP*_1_	To be sent to *TP*_2_
					*C*_*a*1_ = *X*_1_ ⊕ ${X}_{1}^{\text{'}}$	*X*_12_
0	0	0	1	1	1	0
0	0	0	1	1	1	0
0	0	0	1	1	1	0
1	1	0	1	0	0	0
1	1	0	1	0	0	0
0	0	0	1	1	1	0
1	1	0	1	0	0	0
1	1	0	1	0	0	0
0	0	0	1	1	1	0
1	0	1	0	0	1	1
0	0	0	1	1	1	0
0	1	1	0	1	0	1
1	1	0	1	0	0	0
1	0	1	0	0	1	1
1	1	0	1	0	0	0
0	1	1	0	1	0	1
1	0	1	0	0	1	1
0	0	0	1	1	1	0
1	0	1	0	0	1	1
1	1	0	1	0	0	0
0	1	1	0	0	0	1
0	0	0	1	1	1	0
0	1	1	0	1	0	1
1	1	0	1	0	0	0
1	0	1	0	0	1	1
1	0	1	0	0	1	1
1	0	1	0	0	1	1
0	1	1	0	1	0	1
0	1	1	0	1	0	1
0	0	1	1	1	1	1
0	1	1	0	1	0	1
0	1	1	0	1	0	1

Here, we provide the necessary equations to verify the equality check by *TP*_1_ and *TP*_2_ for the various suggested protocols.

### Two-party QPC with two rounds

From Eqs. (11) and (12), *TP*_1_ computes

$$\begin{array}{rcl}{R}_{1} & = & {C}_{a1}\oplus {C}_{b1},\\  & = & {X}_{1}\oplus {X}_{1}^{\text{'}}\oplus {Y}_{1}\oplus {Y}_{1}^{\text{'}},\\  & = & {K}_{rand}^{1}\oplus {X}_{part\_1}\oplus {X}_{1}^{\text{'}}\oplus {K}_{rand}^{1}\oplus {Y}_{part\_1}\oplus {Y}_{1}^{\text{'}}\\  & = & {X}_{part\_1}\oplus {X}_{1}^{\text{'}}\oplus {Y}_{part\_1}\,\oplus {Y}_{1}^{\text{'}}.\end{array}$$

From Eqs. (13) and (14), *TP*_2_ computes

$$\begin{array}{rcl}{R}_{2} & = & {X}_{12}\oplus {Y}_{12},\\  & = & {X}_{1}\oplus {X}_{2}\oplus {Y}_{1}\oplus {Y}_{2},\\  & = & {K}_{rand}^{1}\oplus {X}_{part\_1}\oplus {K}_{rand}^{2}\oplus {X}_{part\_2}\oplus {K}_{rand}^{1}\oplus {Y}_{part\_1}\oplus {K}_{rand}^{2}\oplus {Y}_{part\_2}.\\  & = & {X}_{part\_1}\oplus {X}_{part\_2}\oplus {Y}_{part\_1}\oplus {Y}_{part\_2}.\end{array}$$

In the proposed protocol, computing only *R*_2_ is not sufficient for getting the comparison result. For example, if we have *X*_1_ = *X*_2_ = 0, *Y*_1_ = *Y*_2_ = 1, and ${K}_{rand}^{1}={K}_{rand}^{2}=0$. Then *R*_2_ = 0 ⊕ 0 ⊕ 1 ⊕ 1 = 0. This means that *X* and *Y* are equal in contrast to the correct comparison result (*R* = *R*_1_ + *R*_2_ = 1 + 0 = 1). In such a case, *R*_1_ guarantees the correctness of the final result.

### MDQPC with two rounds

From Eq. (15), for *M* = 3, *TP*_1_ computes

$$\begin{array}{rcl}{R}_{1} & = & ({C}_{1,1}\oplus {C}_{2,1})+({C}_{2,1}\oplus {C}_{3,1}).\\ {R}_{1} & = & ({X}_{1,1}\oplus {X}_{1,1}^{\text{'}}\oplus {X}_{2,1}\oplus {X}_{2,1}^{\text{'}})+({X}_{2,1}\oplus {X}_{2,1}^{\text{'}}\oplus {X}_{3,1}\oplus {X}_{3,1}^{\text{'}}).\\ {R}_{1} & = & ({K}_{rand}^{1}\oplus {X}_{1,part\_1}\oplus {X}_{1,1}^{\text{'}}\oplus {K}_{rand}^{1}\oplus {X}_{2,part\_1}\oplus {X}_{2,1}^{\text{'}})\\  &  & +\,({K}_{rand}^{1}\oplus {X}_{2,part\_1}\oplus {X}_{2,1}^{\text{'}}\oplus {K}_{rand}^{1}\oplus {X}_{3,part\_1}\oplus {X}_{3,1}^{\text{'}}).\end{array}$$

From Eq. (16), for *M* > 3, *TP*_1_ computes

$$\begin{array}{rcl}{R}_{1} & = & ({C}_{1,1}\oplus {C}_{2,1})+({C}_{2,1}\oplus {C}_{3,1})+\cdots +({C}_{M-1,1}\oplus {C}_{M,1}),\\ {R}_{1} & = & ({X}_{1,1}\oplus {X}_{1,1}^{\text{'}}\oplus {X}_{2,1}\oplus {X}_{2,1}^{\text{'}})+({X}_{2,1}\oplus {X}_{2,1}^{\text{'}}\oplus {X}_{3,1}\oplus {X}_{3,1}^{\text{'}})+\cdots \\  &  & +\,({X}_{M-1,1}\oplus {X}_{M-1,1}^{\text{'}}\oplus {X}_{M,1}\oplus {X}_{M,1}^{\text{'}}),\\ {R}_{1} & = & ({K}_{rand}^{1}\,\oplus \,{X}_{1,part\_1}\oplus {X}_{1,1}^{\text{'}}\oplus {K}_{rand}^{1}\,\oplus {X}_{2,part\_1}\oplus {X}_{2,1}^{\text{'}})\\  &  & +\,({K}_{rand}^{1}\oplus {X}_{2,part\_1}\oplus {X}_{2,1}^{\text{'}}\oplus {K}_{rand}^{1}\oplus {X}_{3,part\_1}\oplus {X}_{3,1}^{\text{'}})\\  &  & +\,\cdots +({K}_{rand}^{1}\oplus {X}_{M-1,part\_1}\oplus {X}_{M-1,1}^{\text{'}}\oplus {K}_{rand}^{1}\oplus {X}_{M,part\_1}\oplus {X}_{M,1}^{\text{'}}).\end{array}$$

In addition, from Eq. (17), for *M* = 3, *TP*_2_ computes

$$\begin{array}{rcl}{R}_{2} & = & ({X}_{1,12}\oplus {X}_{2,12})+({X}_{2,12}\oplus {X}_{3,12}),\\ {R}_{2} & = & ({X}_{1,1}\oplus {X}_{1,2}\oplus {X}_{2,1}\oplus {X}_{2,2})+({X}_{2,1}\oplus {X}_{2,2}\oplus {X}_{3,1}\oplus {X}_{3,2}),\\ {R}_{2} & = & ({K}_{rand}^{1}\oplus {X}_{1,par{t}_{1}}\oplus {K}_{rand}^{2}\oplus {X}_{1,par{t}_{2}}\oplus {K}_{rand}^{1}\oplus {X}_{2,par{t}_{1}}\oplus {K}_{rand}^{2}\oplus {X}_{2,par{t}_{2}})\\  &  & +\,({K}_{rand}^{1}\oplus {X}_{2,par{t}_{1}}\oplus {K}_{rand}^{2}\oplus {X}_{2,par{t}_{2}}\oplus {K}_{rand}^{1}\oplus {X}_{3,par{t}_{1}}\oplus {K}_{rand}^{2}\oplus {X}_{3,par{t}_{2}}),\end{array}$$

where ${K}_{rand}^{1}$ and ${K}_{rand}^{2}$ represent the random encryption keys for the first and second parts of the private information. *X*_*i*,*part*_1_ and *X*_*i*,*part*_2_ represent the first part and second part of the private information of *P*_*i*_.

From Eq. (18), for *M* > 3, *TP*_2_ computes

$$\begin{array}{rcl}{R}_{2} & = & ({X}_{1,12}\oplus {X}_{2,12})+({X}_{2,12}\oplus {X}_{3,12})+\cdots +({X}_{M-1,12}\oplus {X}_{M,12}).\\ {R}_{2} & = & ({X}_{1,1}\oplus {X}_{1,2}\oplus {X}_{2,1}\oplus {X}_{2,2})+({X}_{2,1}\oplus {X}_{2,2}\oplus {X}_{3,1}\oplus {X}_{3,2})\\  &  & +\,\cdots +({X}_{M-1,1}\oplus {X}_{M-1,2}\oplus {X}_{M,1}\oplus {X}_{M,2}),\\ {R}_{2} & = & ({K}_{rand}^{1}\,\oplus {X}_{1,part\_1}\oplus {K}_{rand}^{2}\oplus {X}_{1,part\_2}\oplus {K}_{rand}^{1}\,\oplus {X}_{2,part\_1}\oplus {K}_{rand}^{2}\,\oplus {X}_{2,part\_2})\\  &  & +\,({K}_{rand}^{1}\,\oplus {X}_{2,part\_1}\oplus {K}_{rand}^{2}\,\oplus {X}_{2,part\_2}\oplus {K}_{rand}^{1}\,\oplus {X}_{3,part\_1}\oplus {K}_{rand}^{2}\,\oplus {X}_{3,part\_2})\\  &  & +\,\cdots +({K}_{rand}^{1}\oplus {X}_{M-1,part\_1}\oplus {K}_{rand}^{2}\oplus {X}_{M-1,part\_2}\oplus {K}_{rand}^{1}\oplus {X}_{M,part\_1}\oplus \,{K}_{rand}^{2}\oplus {X}_{M,part\_2}).\end{array}$$

Thus, if *R*_1_ = 0 and *R*_2_ = 0, *R* = *R*_1_ + *R*_2_ = 0, hence *X*_1_, *X*_2_, ..., *X*_*M*_ are equal. Otherwise, *X*_1_, *X*_2_, ..., *X*_*M*_ are not equal.

### MDQPC with B-block

From Eq. (20), for *M* = 3, *TP*_1_ computes

$$\begin{array}{rcl}{R}_{1}^{1} & = & ({C}_{1,1}\oplus {C}_{2,1})+({C}_{2,1}\oplus {C}_{3,1})({R}_{1}^{2}=({C}_{1,2}\oplus {C}_{2,2})\\  &  & +\,({C}_{2,2}\oplus {C}_{3,2}),\,\ldots ,\,{R}_{1}^{B}=({C}_{1,B}\oplus {C}_{2,B})+({C}_{2,B}\oplus {C}_{3,B})),\end{array}$$

So,

$$\begin{array}{rcl}{R}_{1}^{1} & = & ({X}_{1,1}\oplus {X}_{1,1}^{\text{'}}\oplus {X}_{2,1}\oplus {X}_{2,1}^{\text{'}})+({X}_{2,1}\oplus {X}_{2,1}^{\text{'}}\oplus {X}_{3,1}\oplus {X}_{3,1}^{\text{'}})\\  &  & \times \,({R}_{1}^{2}=({X}_{1,2}\oplus {X}_{1,2}^{\text{'}}\oplus {X}_{2,2}\oplus {X}_{2,2}^{\text{'}})+({X}_{2,2}\oplus {X}_{2,2}^{\text{'}}\oplus {X}_{3,2}\oplus {X}_{3,2}^{\text{'}}),\,\ldots ,\\  &  & {R}_{1}^{B}=({X}_{1,B}\oplus {X}_{1,B}^{\text{'}}\oplus {X}_{2,B}\oplus {X}_{2,B}^{\text{'}})+({X}_{2,B}\oplus {X}_{2,B}^{\text{'}}\oplus {X}_{3,B}\oplus {X}_{3,B}^{\text{'}})),\\ {R}_{1}^{1} & = & ({K}_{rand}^{1}\oplus {X}_{1,part\_1}\oplus {X}_{1,1}^{\text{'}}\oplus {K}_{rand}^{1}\oplus {X}_{2,part\_1}\oplus {X}_{2,1}^{\text{'}})\\  &  & +\,({K}_{rand}^{1}\oplus {X}_{2,part\_1}\oplus {X}_{2,1}^{\text{'}}\oplus {K}_{rand}^{1}\oplus {X}_{3,part\_1}\oplus {X}_{3,1}^{\text{'}})\\  &  & \times ({R}_{1}^{2}=({K}_{rand}^{1,,2}\oplus {X}_{1,part\_2}\oplus {X}_{1,2}^{\text{'}}\oplus {K}_{rand}^{1,2}\oplus {X}_{2,part\_2}\oplus {X}_{2,2}^{\text{'}})\\  &  & +\,({K}_{rand}^{1,2}\oplus {X}_{2,part\_2}\oplus {X}_{2,2}^{\text{'}}\oplus {K}_{rand}^{1,2}\oplus {X}_{3,part\_2}\oplus {X}_{3,2}^{\text{'}}),\,\ldots ,\\  &  & {R}_{1}^{B}=({K}_{rand}^{1,{\rm{B}}}\oplus {X}_{1,part\_{\rm{B}}}\oplus {X}_{1,B}^{\text{'}}\oplus {K}_{rand}^{1,{\rm{B}}}\oplus {X}_{2,part\_{\rm{B}}}\oplus {X}_{2,B}^{\text{'}})\\  &  & +\,({K}_{rand}^{1,{\rm{B}}}\oplus {X}_{2,part\_{\rm{B}}}\oplus {X}_{2,B}^{\text{'}}\oplus {K}_{rand}^{1,{\rm{B}}}\oplus {X}_{3,part\_{\rm{B}}}\oplus {X}_{3,B}^{\text{'}})).\end{array}$$

For *M* > 3,

$$\begin{array}{rcl}{R}_{1}^{1} & = & ({C}_{1,1}\oplus {C}_{2,1})+({C}_{2,1}\oplus {C}_{3,1})+\ldots +({C}_{M-1,1}\oplus {C}_{M,1})({R}_{1}^{2}=({C}_{1,2}\oplus {C}_{2,2})\\  &  & +\,({C}_{2,2}\oplus {C}_{3,2})+\ldots +({C}_{M-1,2}\oplus {C}_{M,2}),\,\ldots ,\,{R}_{M}^{B}=({C}_{1,B}\oplus {C}_{2,B})\\  &  & +\,({C}_{2,B}\oplus {C}_{3,B})+\ldots +({C}_{M-1,B-1}\oplus {C}_{M,B})),\end{array}$$

So,

$$\begin{array}{rcl}{R}_{1}^{1} & = & ({X}_{1,1}\oplus {X}_{1,1}^{\text{'}}\oplus {X}_{2,1}\oplus {X}_{2,1}^{\text{'}})+({X}_{2,1}\oplus {X}_{2,1}^{\text{'}}\oplus {X}_{3,1}\oplus {X}_{3,1}^{\text{'}})\\  &  & +\,\ldots +({X}_{M-1,1}\oplus {X}_{M-1,1}^{\text{'}}\oplus {X}_{M,1}\oplus {X}_{M,1}^{\text{'}})\\  &  & \times ({R}_{1}^{2}=\,({X}_{1,2}\oplus {X}_{1,2}^{\text{'}}\oplus {X}_{2,2}\oplus {X}_{2,2}^{\text{'}})+({X}_{2,2}\oplus {X}_{2,2}^{\text{'}}\oplus {X}_{3,2}\oplus {X}_{3,2}^{\text{'}})+\ldots \\  &  & +\,({X}_{M-1,2}\oplus {X}_{M-1,2}^{\text{'}}\oplus {X}_{M,2}\oplus {X}_{M,2}^{\text{'}}),\,\ldots ,\,{R}_{M}^{B}=({X}_{1,B}\oplus {X}_{1,B}^{\text{'}}\oplus {X}_{2,B}\oplus {X}_{2,B}^{\text{'}})\\  &  & +\,({X}_{2,B}\oplus {X}_{2,B}^{\text{'}}\oplus {X}_{3,B}\oplus {X}_{3,B}^{\text{'}})+\ldots +({X}_{M-1,B}\oplus {X}_{M-1,B}^{\text{'}}\oplus {X}_{M,B}\oplus {X}_{M,B}^{\text{'}})),\\ {R}_{1}^{1} & = & ({K}_{rand}^{1,1}\oplus {X}_{1,part\_1}\oplus {X}_{1,1}^{\text{'}}\oplus {K}_{rand}^{1,1}\oplus {X}_{2,part\_1}\oplus {X}_{2,1}^{\text{'}})\\  &  & +\,({K}_{rand}^{1,1}\oplus {X}_{2,part\_1}\oplus {X}_{2,1}^{\text{'}}\oplus {K}_{rand}^{1,1}\oplus {X}_{3,part\_1}\oplus {X}_{3,1}^{\text{'}})+\ldots \\  &  & +\,({K}_{rand}^{1,1}\,\oplus {X}_{M-1,part\_1}\oplus {X}_{M-1,1}^{\text{'}}\oplus {K}_{rand}^{1,1}\oplus {X}_{M,part\_1}\oplus {X}_{M,1}^{\text{'}})\\  &  & \times ({R}_{1}^{2}=({K}_{rand}^{1,2}\oplus {X}_{1,part\_2}\oplus {X}_{1,2}^{\text{'}}\oplus {K}_{rand}^{1,2}\oplus {X}_{2,part\_2}\oplus {X}_{2,2}^{\text{'}})\\  &  & +\,({K}_{rand}^{1,2}\oplus {X}_{2,part\_1}\oplus {X}_{2,2}^{\text{'}}\oplus {K}_{rand}^{1,2}\oplus {X}_{3,part\_2}\oplus {X}_{3,2}^{\text{'}})+\ldots \\  &  & +\,({K}_{rand}^{1,2}\,\oplus {X}_{M-1,part\_2}\oplus {X}_{M-1,2}^{\text{'}}\oplus {K}_{rand}^{1,2}\,\oplus {X}_{M,part\_2}\oplus {X}_{M,2}^{\text{'}}),\,\ldots ,\\  &  & {R}_{M}^{B}=({K}_{rand}^{1,B}\oplus {X}_{1,part\_{\rm{B}}}\oplus {X}_{1,B}^{\text{'}}\oplus {K}_{rand}^{1,B}\oplus {X}_{2,part\_{\rm{B}}}\oplus {X}_{2,B}^{\text{'}})\\  &  & +\,({K}_{rand}^{1,B}\oplus {X}_{2,part\_{\rm{B}}}\oplus {X}_{2,B}^{\text{'}}\oplus {K}_{rand}^{1,B}\oplus {X}_{3,part\_{\rm{B}}}\oplus {X}_{3,B}^{\text{'}})+\ldots \\  &  & +\,({K}_{rand}^{1,B}\oplus {X}_{M\_1,part\_{\rm{B}}}\oplus {X}_{M-1,B}^{\text{'}}\oplus {K}_{rand}^{1,B}\oplus {X}_{M,part\_{\rm{B}}}\oplus {X}_{M,B}^{\text{'}})),\end{array}$$

In addition, from Eq. (22), for *M* = 3, *TP*_2_ computes

$$\begin{array}{rcl}{R}_{2}^{1} & = & ({X}_{12}^{1,1}\oplus {X}_{12}^{2,1})+({X}_{12}^{2,1}\oplus {X}_{12}^{3,1})({R}_{2}^{2}=({X}_{12}^{1,2}\oplus {X}_{12}^{2,2})\\  &  & +\,({X}_{12}^{2,2}\oplus {X}_{12}^{3,2}),\,\ldots ,\,{R}_{2}^{B}=({X}_{12}^{1,B}\oplus {X}_{12}^{2,B})+({X}_{12}^{2,B}\oplus {X}_{12}^{3,B})),\\ {R}_{2}^{1} & = & ({X}_{1}^{1,1}\oplus {X}_{2}^{1,1}\oplus {X}_{1}^{2,1}\oplus {X}_{2}^{2,1})+\,({X}_{1}^{2,1}\oplus {X}_{2}^{2,1}\oplus \,{X}_{1}^{3,1}\oplus \,{X}_{2}^{3,1})\\  &  & \times ({R}_{2}^{2}=({X}_{1}^{1,2}\oplus {X}_{2}^{1,2}\oplus {X}_{1}^{2,2}\oplus {X}_{2}^{2,2})+({X}_{1}^{2,2}\oplus {X}_{2}^{2,2}\oplus {X}_{1}^{3,2}\oplus {X}_{2}^{3,2}),\ldots ,\\ {R}_{2}^{B} & = & ({X}_{1}^{1,B}\oplus {X}_{2}^{1,B}\oplus {X}_{1}^{2,B}\oplus {X}_{2}^{2,B})+({X}_{1}^{2,B}\oplus {X}_{2}^{2,B}\oplus {X}_{1}^{3,B}\oplus {X}_{2}^{3,B})),\end{array}$$

$$\begin{array}{rcl}{R}_{2}^{1} & = & ({K}_{rand}^{1,1}\oplus {X}_{part\_1}^{1,1}\oplus {K}_{rand}^{2,1}\oplus {X}_{part\_2}^{1,1}\oplus {K}_{rand}^{1,1}\oplus {X}_{part\_1}^{2,1}\oplus {K}_{rand}^{2,1}\oplus {X}_{part\_2}^{2,1})\\  &  & +\,({K}_{rand}^{1,1}\oplus {X}_{part\_1}^{2,1}\oplus {K}_{rand}^{2,1}\oplus {X}_{part\_2}^{2,1}\oplus \,{K}_{rand}^{1,1}\oplus {X}_{part\_1}^{3,1}\oplus {K}_{rand}^{2,1}\oplus {X}_{part\_2}^{3,1})\\  &  & \times ({R}_{2}^{2}=({K}_{rand}^{1,2}\oplus {X}_{part\_1}^{1,2}\oplus {K}_{rand}^{2,2}\oplus {X}_{part\_2}^{2,2}\oplus {K}_{rand}^{1,2}\oplus {X}_{part\_1}^{2,2}\oplus {K}_{rand}^{2,2}\oplus {X}_{part\_2}^{2,2})\\  &  & +\,({K}_{rand}^{1,2}\oplus {X}_{part\_1}^{2,2}\oplus {K}_{rand}^{2,2}\oplus {X}_{part\_2}^{2,2}\oplus {K}_{rand}^{1,3}\oplus {X}_{part\_1}^{3,2}\oplus {K}_{rand}^{2,3}\oplus {X}_{part\_2}^{3,2}),\,\ldots ,\\  &  & {R}_{2}^{B}=({K}_{rand}^{1,{\rm{B}}}\oplus {X}_{part\_1}^{1,B}\oplus {K}_{rand}^{2,{\rm{B}}}\oplus {X}_{part\_2}^{1,B}\oplus {K}_{rand}^{1,{\rm{B}}}\oplus {X}_{part\_1}^{2,B}\oplus {K}_{rand}^{2,{\rm{B}}}\oplus {X}_{part\_2}^{2,B})\\  &  & +\,({K}_{rand}^{1,{\rm{B}}}\oplus {X}_{part\_1}^{2,B}\oplus {K}_{rand}^{2,{\rm{B}}}\oplus {X}_{part\_2}^{2,B}\oplus {K}_{rand}^{1,{\rm{B}}}\oplus {X}_{part\_1}^{3,B}\oplus {K}_{rand}^{2,{\rm{B}}}\oplus {X}_{part\_2}^{3,B})),\end{array}$$

where ${{\rm{K}}}_{rand}^{1,\,j}$ and ${{\rm{K}}}_{rand}^{2,\,j}$ are random subkeys for encrypting the first and second part of the *jth* block, *j* = 1, 2, …, *B*.

From Eq. (23) for *M* > 3, *TP*_2_ computes

$$\begin{array}{rcl}{R}_{2}^{1} & = & ({X}_{12}^{1,1}\oplus {X}_{12}^{2,1})+({X}_{12}^{2,1}\oplus {X}_{12}^{3,1})+\ldots +({X}_{12}^{M-1,1}\oplus {X}_{12}^{M,1})(\ldots ,\\  &  & {R}_{2}^{B}=({X}_{12}^{1,B}\oplus {X}_{12}^{2,B})+({X}_{12}^{2,B}\oplus {X}_{12}^{3,B})+\ldots +({X}_{12}^{M-1,B}\oplus {X}_{12}^{M,B})),\end{array}$$

So, we can get

$$\begin{array}{rcl}{R}_{2}^{1} & = & ({X}_{1}^{1,1}\oplus {X}_{2}^{1,1}\oplus {X}_{1}^{2,1}\oplus {X}_{2}^{2,1})+({X}_{1}^{2,1}\oplus {X}_{2}^{2,1}\oplus {X}_{1}^{3,1}\oplus {X}_{2}^{3,1})+\ldots \\  &  & +\,({X}_{1}^{M-1,1}\oplus {X}_{2}^{M-1,1}\oplus {X}_{1}^{M,1}\oplus {X}_{2}^{M,1})(\ldots ,\,{R}_{2}^{B}=({X}_{1}^{1,B}\oplus {X}_{2}^{1,B}\oplus {X}_{1}^{2,B}\oplus {X}_{2}^{2,B})\\  &  & +\,({X}_{1}^{2,B}\oplus {X}_{2}^{2,B}\oplus {X}_{1}^{3,B}\oplus {X}_{2}^{3,B})+\ldots +({X}_{1}^{M-1,B}\oplus {X}_{2}^{M-1,B}\oplus {X}_{1}^{M,B}\oplus {X}_{2}^{M,B})),\end{array}$$

$$\begin{array}{rcl}{R}_{2}^{1} & = & ({K}_{rand}^{1,1}\oplus {X}_{part\_1}^{1,1}\oplus {K}_{rand}^{2,1}\oplus {X}_{part\_2}^{1,1}\oplus {K}_{rand}^{1,1}\oplus {X}_{part\_1}^{2,1}\oplus {K}_{rand}^{2,1}\oplus {X}_{part\_2}^{2,1})\\  &  & +\,({K}_{rand}^{1,1}\oplus {X}_{part\_1}^{2,1}\oplus {K}_{rand}^{2,1}\oplus {X}_{part\_2}^{2,1}\oplus {K}_{rand}^{1,1}\oplus {X}_{part\_1}^{3,1}\oplus {K}_{rand}^{2,1}\oplus {X}_{part\_2}^{3,1})\\  &  & +\,\ldots +({K}_{rand}^{1,1}\oplus {X}_{part\_1}^{M-1,1}\oplus {K}_{rand}^{2,1}\oplus {X}_{part\_2}^{M-1,1}\oplus {K}_{rand}^{1,1}\oplus {X}_{part\_1}^{M,1}\oplus {K}_{rand}^{2,1}\oplus {X}_{part\_2}^{M,1})\\  &  & \times \,(\ldots ,\,{R}_{2}^{B}=({K}_{rand}^{1,{\rm{B}}}\oplus {X}_{part\_1}^{1,B}\oplus {K}_{rand}^{2,{\rm{B}}}\oplus {X}_{part\_2}^{1,B}\oplus {K}_{rand}^{1,{\rm{B}}}\oplus {X}_{part\_1}^{2,B}\oplus {K}_{rand}^{2,{\rm{B}}}\oplus {X}_{part\_2}^{2,B})\\  &  & +\,({K}_{rand}^{1,{\rm{B}}}\oplus {X}_{part\_1}^{2,B}\oplus {K}_{rand}^{2,{\rm{B}}}\oplus {X}_{part\_2}^{2,B}\oplus {K}_{rand}^{1,{\rm{B}}}\oplus {X}_{part\_1}^{3,B}\oplus {K}_{rand}^{2,{\rm{B}}}\oplus {X}_{part\_2}^{3,B})+\ldots \\  &  & +\,({K}_{rand}^{1,{\rm{B}}}\oplus {X}_{part\_11}^{M-1,B}\oplus {K}_{rand}^{2,{\rm{B}}}\oplus {X}_{part\_2}^{M-1,B}\oplus {K}_{rand}^{1,{\rm{B}}}\oplus {X}_{part\_1}^{M,B}\oplus {K}_{rand}^{2,{\rm{B}}}\oplus {X}_{part\_2}^{M,B})).\end{array}$$

Thus, if ${R}_{2}^{1}={R}_{2}^{2}=\cdots ={R}_{2}^{B}=0$, *X*_1_, *X*_2_, …, *X*_*M*_ are equal. Otherwise, *X*_1_, *X*_2_, …, *X*_*M*_ are not equal.

### Security analysis

Here, we will show the robustness of the proposed QPC protocol against insider and outsider attacks. If the length of the secrets is odd, it should be modified. This process not only contributes to correctly executing the proposed protocol but also assists in enhancing the security of the protocol by altering the original secret bits without affecting the final comparison result. Moreover, two random keys are generated and distributed between TPs and participants to encrypt the private information of parties. As discussed in^30,48^, for improving the efficiency of the proposed DMQPC protocol, the private information of parties can be divided into several blocks of data. If the comparison result of a particular block is not equal, *TP*_1_ announces that the outcome of the comparison is not similar; hence there is no need to execute the remaining rounds. The three protocols in subsections 2.1, 2.4, and 2.5 are similar. Also, in the two-party QPC with two rounds, the quantum channel in the first-round is similar to the quantum channel in the second-round, so here we only analyze the quantum communication in the first-round between the participants and *TP*_1_.

### Outside attack

In the two-party situation, Alice (Bob) sends ${S}_{a}^{\text{'}}$ (${S}_{b}^{\text{'}}$) to *TP*_1_, protected by single decoy photons *l*_*a*1_ (*l*_*b*1_). Alice (Bob) then announces the measurement bases and the positions of all inserted decoy particles. Subsequently, the *TP*_1_ announces the measurement results of all embedded decoy particles. Alice (Bob) then checks the security of the communication by checking whether the measurement results of the decoy particles are correct. Since the outside attacker does not learn the measurement bases of the decoy particles and their positions ahead of time, the well-known attacks such as entangle-resend attacks^32^, correlation-elicitation attacks^49^, and intercept-resend attacks^50^ can be detected with nonzero probability^51^. For instance, if the eavesdropper, Eve, attempts to measure the decoy photons |0〉 or |1〉 in ${S}_{a}^{\text{'}}$ (${S}_{b}^{\text{'}}$) with the correct basis (e.g., Z-basis), she successfully passes the public eavesdropping check. But, If Eve attempts to measure the decoy photons |0〉 or |1〉 in ${S}_{a}^{\text{'}}$ (${S}_{b}^{\text{'}}$) with an incorrect basis (e.g., X-basis), she will be detected with a probability of 50%. The probability of choosing the wrong measuring basis is 50%. Thus, the rate of detecting Eve for each single decoy photon is 25% (i.e., 50% × 50%). Hence, the rate of detecting Eve for *l* single decoy photon is 1−(3/4)^*l*^, where |*l*| = |*l*_*a*1_| = |*l*_*b*1_|. This rate approaches 1 when *l* is large enough. Furthermore, a Trojan-horse attack^52^ is prevented since photons are transmitted only once from participants to the *TP*_1_. So, our two-party QPC protocol is fully secure against outsider attacks. Since the proposed DMQPC protocol uses the same strategy as the two-party process, it is also secure against outsider attacks.

### Participant’s attack

A significant advantage of our three different scenarios is that participant attacks such as collusion attack and cheating attack are not possible for the proposed protocols. Each participant receives two random keys from *TP*_1_ and *TP*_2_ for encrypting her/his secret without the participation or assistance of other parties. Therefore, there is no exchange of information or even communication among participants, and each participant sends the private information directly to the *TP*_1_ and *TP*_2_ through quantum channels. Thus, to steal confidential information, dishonest participants must adopt Eve’s attack strategies because they act as outside attackers. As discussed above, the protocol is secure against outside attacks.

### TP’s attack

TP’s attack is another type of participant’s attack which could threaten the security of the protocol. Here we prove that our scheme is secure against dishonest or malicious TPs. Firstly, with the assumption that the two TPs are not allowed to collude together or with participants, our protocol is secure since the encrypted data is distributed to two independent TPs for computing the final comparison result. To clarify, assume we have a secret *a* and an encryption key *b* and *c* = *a* ⊕ *b*. The probability of an attacker to know *a* is $\frac{1}{{2}^{n}}$, where *n* is the length of the secret *a*^53^. In the proposed protocol, from *TP*_2_’s point of view, as shown in Table 4, *X*_12_ = *X*_1_ ⊕ *X*_2_. From Eqs. (2) and (3), ${X}_{1}={K}_{rand}^{1}\,\oplus \,{X}_{part\_1}$ and ${X}_{2}={K}_{rand}^{2}\,\oplus \,{X}_{part\_2}$ where *X*_*part*_1_ is the first part of the secret message (*X*) and *X*_*part*_2_ is the second part of *X*. The probability of *TP*_2_ to know *X* is $\frac{1}{{2}^{\frac{n}{2}}}$, where *n* is the length of the secret *X*, and $\frac{n}{2}$ is the length of *X*_12_. When *n* is large enough, the probability of getting the secret data is negligible. In addition, according to Table 4, *TP*_2_ can obtain *X*_12_ = 1 ⊕ ${X}_{1}^{\text{'}}$. Hence, if *X*_12_ = 0 then *TP*_2_ can learn that ${X}_{1}^{\text{'}}$ = 1, otherwise ${X}_{1}^{\text{'}}$ = 0. However, the private information of Alice is still secure against *TP*_2_’s attack for two reasons: (1) *TP*_2_ cannot learn any private information of Alice using ${X}_{1}^{\text{'}}$; (2) the private information of Alice (*X*_*part*_1_ and *X*_*part*_2_) is protected by two random keys (${K}_{rand}^{1}$ and ${K}_{rand}^{2}$).

From *TP*_1_’s point of view, Alice sends her encrypted secret (i.e., *C*_*a*1_ = *X*_1_ ⊕ ${X}_{1}^{\text{'}}$ (*C*_*a*2_ = *X*_2_ ⊕ ${X}_{2}^{\text{'}}$)) to *TP*_1_. *TP*_1_ cannot reveal any useful information without knowing *X*_1_ or ${X}_{1}^{\text{'}}$ (*X*_2_ or ${X}_{2}^{\text{'}}$). The probability of knowing the original secret is $\frac{1}{{2}^{\frac{n}{2}}}$, where *n* is the length of the secret *X*, and $\frac{n}{2}$ is the length of *C*_*a*1_(*C*_*a*2_). When *n* is large enough, the probability of *TP*_1_ to know the original secret is negligible. Also, when participants’ secret data is divided into B blocks, the probability of *TP*_1_(*TP*_2_) to identify the original secret is ${(\frac{1}{{2}^{((\frac{n}{B})/2)}})}^{B}$, where *B* is the number of blocks. In addition, according to Table 4, *TP*_1_ can obtain *C*_*a*1_ = 1 ⊕ *X*_2_ and *X*_2_ = 1 ⊕ *C*_*a*1_. Hence, if *C*_*a*1_ = 0; then *TP*_1_ can learn that *X*_2_ = 1, otherwise *X*_2_ = 0. However, the private information of Alice (*X*_*part*_1_ and *X*_*part*_2_) is still secure against *TP*_1_’s attack, since ${X}_{part\_1}={X}_{1}\oplus {K}_{rand}^{1}$ and ${X}_{part\_2}={X}_{2}\oplus {K}_{rand}^{2}$.

## Efficiency Analysis

The used qubit efficiency is defined as $\eta =\frac{C}{q}$ ^54–56^, where *C* refers to all classical bits that can be transmitted, and *q* refers to the total number of used photons. In the two-party case, the proposed protocol is executed in one or two rounds depending on the first-round result. If the proposed protocol is executed in one round, both Alice and Bob prepare $\frac{n}{2}$ single photons. The protocol is completed in one round when the comparison result of the first parts of Alice’s secret and Bob’s secret are not equal. Thus, the qubit efficiency is $\frac{n}{\frac{n}{2}+\frac{n}{2}}$ (i.e., 100%). However, if the first parts of Alice’s secret and Bob’s secret are equal, the proposed protocol is executed in two rounds. Hence, the qubit efficiency is $\frac{n}{2(\frac{n}{2}+\frac{n}{2})}$ (i.e. 50%). In the multi-party protocol with two rounds, the qubit efficiency of one round is $\frac{n}{M\frac{n}{2}}$, and the qubit efficiency for the two rounds is $\frac{n}{Mn}$. In the multi-party protocol with B blocks, the proposed protocol is executed in one or more blocks depending on the previous block result. Thus, the qubit efficiency is ranging from $\frac{n}{M\,{r}_{n}}$ to $\frac{n}{M\,n}$, where ${r}_{n}=\frac{n}{2B}$ is the number of bits in each round and *B* is the number of determined blocks. For example, consider four participants (*M* = 4) who would like to compare their secrets of length 12 bits (*n* = 12). In this case, they can divide the secret into 2, 3, or 6 blocks, each part containing 6 bits, 4 bits, or 2 bits, respectively. Assume that they choose to divide the secrets into 2 blocks (i.e., *B* = 2) and each block contains 6 bits (i.e., $\frac{n}{B}=6$); hence the ${r}_{n}=\frac{12}{4}=3$. Then the qubit efficiency ranges from 25% to 100%. It should be noted that the qubit efficiency increases or decreases depending on the number of participants and selected blocks. For comparison, in Liu and Wang’s protocol^46^, the qubit efficiency is $\frac{n}{M(\frac{n}{2}+\frac{n}{2})}$, and for *n* = 12 and *M* = 4, the qubit efficiency is equal to 40%.

## Comparison

Here we compare the performance of our DMQPC proposed scheme with previous MQPC schemes. We first compare our DMQPC protocol with Liu and Wang’s protocol^46^ (see Table 6). We then compare our DMQPC protocol with previous MQPC protocols.

**Table 6:** Comparison to Liu-Wang protocol^46^.

Parameters	Liu-Wang protocol^46^	Our protocol
Quantum resource	Single photon states	Single photon states
Number of TPs	One	Two
Secure against participant attack	No	Yes
Quantum measurement (TP)	Single photon measurements	Single photon measurements
Quantum measurement (parties)	Single photon measurements	Single photon measurements
Preparing single photons (TP)	Yes	Yes
Preparing single photons (parties)	Yes	Yes
Dynamic	Yes	Yes
The Flexibility of comparing the private information of parties	TP can compare the secret information of any two parties of *M* (*M* ≥ 4) parties with the assistance of other *M*−2 parties	TPs can compare the secret information of any subset of *M* parties without any assistance of other parties
Joining and leaving the comparison protocol	Any subset of *M* parties can join in the protocol before the quantum states are measured	Any subset of *M* parties can join in or leave the protocol at any time without any extra conditions
The cost of transmission	All private information of parties should be transmitted among parties for deducing the final result of the comparison	In case of executing the protocol in one round, only the first part of the secret bits is transmitted to *TP*_1_ for deducing the final result of the comparison

Abulkasim *et al*.^57^ showed that the Liu-Wang protocol suffers from participant attack. In our proposed protocol, participant attack is not possible. Thus, our protocol is safe not only against well-known participant attacks but also against potential participant attacks. Both the Liu-Wang protocol and our protocol use single photon states as a quantum resource and perform single photon measurements. The Liu-Wang protocol uses one TP who performs single photon measurements. In our protocol, two TPs are adopted and they also perform single photon preparation and measurements.

Like the Liu-Wang protocol, in our scheme, both the TP and the participants prepare single photons for deducing the comparison result. Like the Liu-Wang protocol, our protocol is dynamic so that any new subset of *M* parties can join or leave the protocol at any time. However, in the Liu-Wang protocol, new participants have to participate in the protocol before the quantum states are measured. Unlike the Liu-Wang protocol, in our scheme, the TPs can compare the private information of any subset of M parties without any assistance from other parties. In contrary to the Liu-Wang protocol, our scheme reduces the cost of communication by half, in some situations, where the protocol can be executed in one round to get the final comparison result.

From Table 7, like the protocols in refs. ^31,40,42–45,58^, our protocol is secure against participant attack. In contrast with the proposed protocols in refs. ^31,40–46^, which suppose that there is a semi-honest TP who executes the QPC protocol loyally, our proposed protocol allows for almost-dishonest TPs. Unlike the protocols in refs. ^31,40–46,58^, our protocol is secure against a malicious *TP*_1_(*TP*_2_). Like the protocols in refs. ^31,46^, our protocol works in an environment where participants and TPs could be strangers, where there is no need for authenticated channels to prevent secret information from leaking. Compared to previous work, our main contribution is that participant attack is not possible in this work, since there is no exchange of information or even communication among participants. In addition, our scheme reduces the cost of communication.

**Table 7:** Comparison to some existing QPC protocols.

Features	Ref. ^58^	Ref. ^40^	Ref. ^41^	Ref. ^42^	Ref. ^43^	Ref. ^44^	Ref. ^31^	Ref. ^45^	Ref. ^46^	Our
Multiparty	No	Yes	Yes	Yes	Yes	Yes	Yes	Yes	Yes	Yes
Dynamic	No	No	No	No	No	No	No	No	Yes	Yes
Secure against participant attack	Yes	Yes	No	Yes	Yes	Yes	Yes	Yes	No	Yes
Secure against the malicious TP	No	No	No	No	No	No	No	No	No	Yes
Work in strangers’ environment^31^	No	No	No	No	No	No	Yes	No	Yes	Yes

## Conclusion

This work proposes a novel dynamic multiparty quantum private comparison protocol that does not allow participant attack. The proposed protocol divides the private information into equal parts, and every participant independently encrypts her/his secrets using two random keys before sending them to two third parties using quantum channels. The protocol is executed in one or more rounds depending on the result of the previous round. The private information can also be divided into a number of blocks, with each block containing two equal parts of the secret. The dynamic nature of the proposed protocol enables the two TPs to compare the private information of any subset of *M* parties without any assistance from other parties. Any subset of *M* parties can join in or leave the protocol at any time without any extra conditions. Our analysis proves that the proposed protocol is correct and fully secure against outside attack. Furthermore, the scheme is not open to participant attacks. Compared to existing schemes, our protocol is more efficient, more secure and more feasible. Thus, our scheme is an ideal choice for comparing private information of *M* parties.
